# Utility of the Triglyceride and HDL Cholesterol Index in Older Persons in Colombia

**DOI:** 10.1002/hsr2.72769

**Published:** 2026-07-09

**Authors:** Carolina Murgueitio Guzmán, Jesús Andrés Beltrán España, Ángel Alberto García Peña, Edward Cáceres, Cándida Díaz‐Brochero, Miguel Ángel Narváez Chaves, Luciano Villa Miranda

**Affiliations:** ^1^ Department of Internal Medicine Pontificia Universidad Javeriana Bogotá Colombia; ^2^ Department of Internal Medicine Hospital Universitario San Ignacio Bogotá Colombia; ^3^ Faculty of Medicine Pontificia Universidad Javeriana Bogotá Colombia; ^4^ Cardiology Unit, Hospital Universitario San Ignacio Universidad Javeriana Bogotá Colombia; ^5^ Department of Epidemiology and Biostatistics Pontificia Universidad Javeriana Bogotá Colombia

**Keywords:** cardiovascular risk, older adult, triglyceride‐to‐HDL ratio

## Abstract

**Background:**

Alterations in lipid metabolism are key contributors to the development of atherogenesis and the increasing prevalence of atherosclerotic cardiovascular diseases, including coronary artery disease, peripheral arterial disease, and cerebrovascular disease. The triglyceride‐to‐HDL cholesterol (TG/HDL‐c) index is commonly used to assess cardiovascular disease risk; however, its effectiveness in older adults remains unclear.

**Methods:**

This study evaluated the association and predictive capacity of the TG/HDL‐c index for cardiometabolic risk factors in older adults from the SABE Colombian study. A cross‐sectional analysis was performed among adults aged ≥ 60 years from the national SABE survey. Univariable and multivariable logistic regression models were constructed to examine associations between the TG/HDL‐c index and diabetes or metabolic syndrome, adjusting for age, sex, BMI, smoking, diet, and physical activity. Discriminatory capacity was assessed using receiver operating characteristic curves, and optimal cut‐off points were determined using the Youden index.

**Results:**

A total of 23,694 participants with a median age of 69 years were included; 57.3% were women. The TG/HDL‐c index was independently associated with both diabetes (adjusted OR per 1 SD: 1.25; 95% CI: 1.13–1.37; *p* < 0.001) and metabolic syndrome (adjusted OR per 1 SD: 2.82; 95% CI: 2.46–3.22; *p* < 0.001). The index demonstrated a good discriminatory performance for diabetes (AUC = 0.89; 95% CI: 0.86–0.93; cut‐off = 9.17; sensitivity = 0.81; specificity = 0.90) and metabolic syndrome (AUC = 0.86; 95% CI: 0.85–0.88; cut‐off = 8.81; sensitivity = 0.71; specificity = 0.92). In contrast, discriminatory performance was limited for hypertension, prediabetes, cardiovascular disease, and cerebrovascular disease (AUC range 0.52–0.67).

**Conclusions:**

The TG/HDL‐c index was independently associated with diabetes and metabolic syndrome and demonstrated good discriminatory performance in this nationally representative sample of older adults. Although these results highlight its potential utility for identifying individuals at higher cardiometabolic risk, further longitudinal studies are needed to validate these findings and determine the prognostic value of TG/HDL‐c for clinical decision‐making in elderly populations.

## Summary

- The TG/HDL‐c index is a simple and inexpensive biomarker for cardiometabolic risk assessment.
- In older Colombian adults, the TG/HDL‐c index demonstrated good discriminatory performance for diabetes and metabolic syndrome but limited performance for other cardiovascular outcomes.
- The TG/HDL‐c index may represent a practical, low‐cost tool to support cardiometabolic risk stratification in routine clinical practice.

## Why Does This Paper Matter?

This study aimed to evaluate the discriminatory performance of the TG/HDL‐c index for cardiovascular risk factors in older adults participating in the SABE Colombia study. We conducted a cross‐sectional analysis of adults aged 60 years and above from the SABE Colombia national survey, which covered 244 municipalities across all departments of the country. The discriminative capacity of the TG/HDL‐c index was further evaluated via ROC curves to identify optimal cut‐off points for these risk factors.

## 1 Background

Cardiovascular disease (CVD) remains the leading cause of death worldwide, accounting for approximately 20.5 million deaths annually and representing a major public health challenge, particularly in low‐ and middle‐income countries where cardiometabolic risk factors are highly prevalent [1, 2]. Alterations in lipid metabolism play a central role in atherogenesis and the development of coronary artery disease, peripheral arterial disease, cerebrovascular disease, and metabolic syndrome. Elevated triglyceride (TG) levels and reduced high‐density lipoprotein cholesterol (HDL‐c) concentrations are hallmarks of atherogenic dyslipidemia, a condition closely associated with insulin resistance, chronic inflammation, and visceral adiposity. Consequently, the TG‐to‐HDL cholesterol (TG/HDL‐c) index has emerged as a simple and inexpensive biomarker associated with endothelial dysfunction, subclinical atherosclerosis, and increased cardiovascular risk [2, 3].

Although other lipid‐related indices, such as the triglyceride–glucose (TyG) index and the LDL/HDL cholesterol ratio, have also been proposed for cardiometabolic risk assessment [4, 5, 6], the TG/HDL‐c index has the practical advantage of relying exclusively on routinely available lipid measurements, facilitating its implementation in clinical practice and large‐scale epidemiological studies [7]. Previous studies have demonstrated that the TG/HDL‐c index effectively identifies metabolic syndrome and other cardiometabolic risk factors, although the optimal cut‐off values vary across populations [8].

In older adults, the TG/HDL‐c index is particularly relevant because aging is associated with progressive insulin resistance, visceral adiposity, chronic low‐grade inflammation, and atherogenic dyslipidemia [9, 10, 11, 12]. These age‐related metabolic alterations increase residual cardiovascular risk, even among individuals with normal LDL‐c concentrations or receiving lipid‐lowering therapy, highlighting the potential value of the TG/HDL‐c index as a complementary biomarker for cardiometabolic risk assessment [2, 9, 10, 11, 12]. However, evidence regarding its discriminatory performance and optimal cut‐off values in older adults remains limited, particularly in Latin American populations. Therefore, this study aimed to evaluate the association and discriminatory performance of the TG/HDL‐c index for cardiometabolic risk factors in a nationally representative sample of older Colombian adults.

## 2 Methods

### 2.1 Design and Context

This study is a secondary analysis of a cross‐sectional survey that included 23,694 older adults and was based on data from the 2015 Survey on Health, Well‐being, and Ageing in Latin America and the Caribbean (SABE) [13]. This multicenter project was conducted by the Pan American Health Organization (PAHO) and supported by the Epidemiological Office of the Ministry of National Health in Bogotá, Colombia. The survey targeted Colombians aged 60 and above, using a combination of purposive and random sampling techniques in both urban and rural areas, ensuring a comprehensive representation of the older adult population. Most participants resided in private homes, with sampling segments determined according to municipal cartography [14]. Ethical approval for the study was obtained from the institutional ethics and research committee of the Pontificia Universidad Javeriana (CIE‐1094‐23). Since this study is a secondary analysis of an anonymized publicly available dataset (SABE Colombia 2015), individual informed consent was not required. All procedures were conducted in accordance with the ethical standards of the institutional research committee and with the 1964 Helsinki Declaration and its later amendments.

### 2.2 Data Collection and Operational Definitions of the Variables

Data collection was carried out by trained personnel from the Universities of Caldas and Valle. Training covered standardized protocols for conducting face–to–face interviews and performing physical measurements to ensure data reliability. Body mass index (BMI) was calculated from measured weight and height and categorized into underweight, normal weight, overweight, and obese groups. Blood pressure was measured via an automatic monitor (OMRON HEM‐705, Omron Healthcare Europe BV, Hoofddorp, Netherlands) following a 5‐min rest period to ensure accuracy.

Venous blood samples were collected after a fasting period of 10–12 h, and biochemical analyses included measurements of HDL‐c, TG, LDL‐c, total cholesterol, and fasting glucose. Data on alcohol consumption, smoking habits, and physical activity were obtained through a standardized proxy questionnaire. Additionally, self‐reported medical conditions, including hypertension, diabetes, and other relevant factors, were documented.

### 2.3 Definition of Metabolic Syndrome

Metabolic syndrome was defined according to the criteria set by the International Diabetes Federation, which include the presence of abdominal obesity, elevated TG levels, reduced HDL‐c levels, high blood pressure, and elevated fasting glucose. This comprehensive definition allowed for the accurate identification of metabolic syndrome among the study participants, aligning with internationally recognized standards [15].

### 2.4 Statistical Analysis

Descriptive statistics were used to summarize sociodemographic characteristics, cardiovascular history, lifestyle factors, cardiovascular risk scores, and metabolic laboratory profiles. Categorical variables are expressed as absolute frequencies and percentages. For continuous variables, measures of central tendency and dispersion were used according to the data distribution: normally distributed variables are presented as the means and standard deviations, whereas nonnormally distributed variables are reported as medians and interquartile ranges.

To evaluate the discriminatory capacity of the TG/HDL‐c index for identifying risk factors such as hypertension, prediabetes, type 2 diabetes mellitus, established CVD, and metabolic syndrome, optimal cut‐off points were determined via the Youden index. This analysis was conducted via receiver operating characteristic (ROC) curves, which enabled the calculation of the area under the curve (AUC), sensitivity, specificity, positive predictive value, and negative predictive value, along with their respective 95% confidence intervals [16]. Statistical analyses were conducted via RStudio software, version 4.4.1, following rigorous standards for statistical evaluation [17, 18].

## 3 Results

The study included 23,694 participants who completed the SABE survey, providing the necessary data for analysis. The general characteristics of the respondents are summarized in Table 1. The median age of the participants was 69 years, with 57.3% being women. Among the participants, 23.5% had arterial hypertension, 16.4% had diabetes mellitus, 13.6% had a history of CVD (including heart failure or infarction), and 4.5% had cerebrovascular disease.

**Table 1:** Characteristics of the population at baseline.

Characteristic	*n* = 23,694
Female sex, *n* (%)	13,582 (57.3)
Age in years, median (IQR)	69 (64–76)
Race, *n* (%)	
White	5215 (22)
Mestizo	8128 (34.3)
Others	657 (2.8)
Not reported	9694 (40.9)
Cardiovascular risk factors, *n* (%)	
Diabetes mellitus	3893 (16.4)
Hypertension	12,690 (53.6)
Respiratory disease	2423 (10.2)
Cardiovascular disease	3234 (13.6)
Cerebrovascular disease	1080 (4.5)
Smoking (former or current), *n* (%)	12,286 (51.9)
BMI (kg/m^2^)	25.5 (22–29)
Lipid profile (median, IQR)	
Total cholesterol (mg/dL)	193 (166–220)
HDL cholesterol (mg/dL)	44 (36–53)
LDL cholesterol (mg/dL)	125 (101–149)
Triglycerides (mg/dL)	141 (105–192)
Glucose (mg/dL)	93 (86–103)
TG/HDL‐c index	8.8 (8.4–9.1)

*Note:* Race – Others (a residual category that groups free responses not classifiable under the previous options, such as Arab, Jewish).

Abbreviations: BMI, body mass index; HDL, high‐density lipoprotein; IQR, interquartile range; LDL, low‐density lipoprotein; TG, triglycerides.

### 3.1 Discriminative Performance of the TG/HDL‐c Index

**Figure 1:**
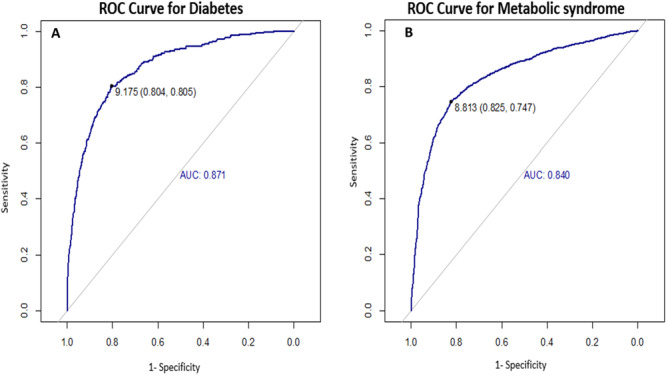
ROC curves of the triglyceride–HDL cholesterol (TG/HDL‐c) index for patients with diabetes (A) and metabolic syndrome (B).

**Figure 2:**
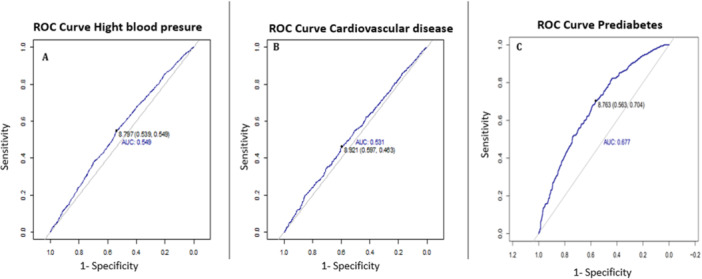
ROC curves of the triglyceride–HDL cholesterol (TG/HDL‐c) index for hypertension (A), cardiovascular disease (B), and prediabetes (C).

The TG/HDL‐c index demonstrated good discriminatory performance in identifying risk factors (see Table 2, Figures 1 and 2). Specifically, the index showed excellent discriminative capacity for diabetes, with an AUC of 0.89 (95% CI: 0.86–0.93) and an optimal cut‐off point of 9.17. For metabolic syndrome, the AUC was 0.86 (95% CI: 0.85–0.88), with a cut‐off point of 8.81, highlighting the index's utility in screening for this condition. The discriminative performance for prediabetes was moderate, with an AUC of 0.67 (95% CI: 0.64–0.71) and a cut‐off point of 8.76. For arterial hypertension, the discriminative ability was limited, with an AUC of 0.52 (95% CI: 0.48–0.55) and a cut‐off point of 8.81. The discriminative capacity of the TG/HDL‐c index was also modest for CVD, with an AUC of 0.53 (95% CI: 0.49–0.58) and a cut‐off point of 8.89, indicating that while the index is a valuable tool for specific conditions, its effectiveness varies depending on the risk factor evaluated.

**Table 2:** Discriminatory performance of the TG/HDL‐c index for cardiovascular risk factors in older adults.

Risk factor	Cut‐off point	Sensitivity (IC 95%)	Specificity (IC 95%)	AUC (IC 95%)	PPV (IC 95%)	NPV (IC 95%)
Prediabetes	8.76	0.71 (0.69–0.73)	0.57 (0.56–0.58)	0.67 (0.64–0.71)	0.35 (0.31–0.39)	0.85 (0.82–0.87)
Diabetes	9.17	0.81 (0.75–0.86)	0.90 (0.87–0.93)	0.89 (0.86–0.93)	0.29 (0.24–0.33)	0.99 (0.98–0.99)
Metabolic syndrome	8.81	0.71 (0.66–0.75)	0.92 (0.89–0.97)	0.86 (0.85–0.88)	0.95 (0.94–0.97)	0.55 (0.51–0.59)
Hypertension	8.81	0.52 (0.51–0.53)	0.52 (0.51–0.53)	0.52 (0.48–0.55)	0.43 (0.39–0.46)	0.53 (0.49–0.57)
Abdominal circumference	8.74	0.63 (0.61–0.65)	0.57 (0.56–0.58)	0.63 (0.61–0.65)	0.65 (0.61–0.68)	0.56 (0.52–0.60)
Cardiovascular disease	8.89	0.50 (0.48–0.52)	0.58 (0.57–0.59)	0.53 (0.49–0.58)	0.16 (0.13–0.19)	0.87 (0.85–0.89)
Cerebrovascular disease	9.12	0.38 (0.35–0.41)	0.74 (0.73–0.75)	0.54 (0.46–0.61)	0.06 (0.04–0.09)	0.96 (0.94–0.97)

Abbreviations: AUC, area under the curve; CI, confidence interval; NPV, negative predictive value; PPV, positive predictive value.

### 3.2 Association Between the TG/HDL‐c Index and Cardiometabolic Outcomes

Univariable and multivariable logistic regression analyses were performed to evaluate the independent association of the TG/HDL‐c index with diabetes and metabolic syndrome (Tables 3 and 4). For diabetes (Table 3), the TG/HDL‐c index was significantly associated in both the univariable (OR per SD: 1.26; 95% CI: 1.15–1.39; *p* < 0.001) and multivariable models (adjusted OR per SD: 1.25; 95% CI: 1.13–1.37; *p* < 0.001), after adjusting for age, sex, BMI, smoking, and physical activity. For metabolic syndrome (Table 4), the association was stronger in both the univariable (OR per SD: 2.65; 95% CI: 2.33–3.00; *p* < 0.001) and multivariable models (adjusted OR per SD: 2.82; 95% CI: 2.46–3.22; *p* < 0.001), confirming the TG/HDL‐c index as an independent marker of cardiometabolic risk in this older adult population.

**Table 3:** Univariable and multivariable logistic regression analyses assessing the association between the TG/HDL‐c index and the presence of diabetes in older adults.

	Univariate model		Multivariate model	
Variable	OR (95% CI)	*p* value	OR (95% CI)	*p* value
TG/HDL‐c (per SD)	1.26 (1.15–1.39)	< 0.001	1.25 (1.13–1.37)	< 0.001
Age (years)	0.98 (0.96–1.00)	0.118	0.99 (0.97–1.01)	0.345
Sex: Women	1.54 (1.15–2.07)	0.004	1.26 (0.92–1.71)	0.146
BMI (kg/m^2^)	1.07 (1.05–1.10)	< 0.001	1.06 (1.04–1.09)	< 0.001
Current smoking	0.70 (0.42–1.19)	0.192	0.86 (0.50–1.47)	0.582
Physical activity: Yes	0.79 (0.60–1.04)	0.092	0.82 (0.62–1.09)	0.167
Fruits/Vegetables ≥ 2/day	1.02 (0.76–1.38)	0.883	—	—

*Note:* AIC = 1501.4.

Abbreviations: BMI, body mass index; CI, confidence interval; OR, odds ratio.

**Table 4:** Univariable and multivariable logistic regression analyses assessing the association between the TG/HDL‐c index and the presence of metabolic syndrome in older adults.

	Univariate model		Multivariate model	
Variable	OR (95% CI)	*p* value	OR (95% CI)	*p* value
TG/HDL‐c (per SD)	2.65 (2.33–3.00)	< 0.001	2.82 (2.46–3.22)	< 0.001
Age (years)	0.99 (0.98–1.00)	0.052	0.99 (0.98–1.00)	0.136
Sex: Women	1.36 (1.14–1.62)	< 0.001	1.22 (1.01–1.48)	0.039
BMI (kg/m^2^)	1.25 (1.22–1.29)	< 0.001	1.23 (1.20–1.27)	< 0.001
Current smoking	0.69 (0.53–0.91)	0.009	0.82 (0.62–1.09)	0.167
Physical activity: Yes	0.64 (0.54–0.76)	< 0.001	0.72 (0.60–0.86)	< 0.001
Fruits/Vegetables ≥ 2/day	0.94 (0.78–1.13)	0.514	—	—

*Note:* AIC = 2380.5.

Abbreviations: BMI, body mass index; CI, confidence interval; OR, odds ratio.

## 4 Discussion

The TG/HDL‐c index demonstrated good discriminatory performance for diabetes and metabolic syndrome, while its ability to identify hypertension, prediabetes, CVD, and cerebrovascular disease was limited. Furthermore, the index remained independently associated with the presence of diabetes and metabolic syndrome after adjustment for potential confounders [2, 11, 19]. To our knowledge, this is the first nationally representative study to evaluate the diagnostic performance of the TG/HDL‐c index in older Colombian adults, supporting its potential role as a simple, inexpensive, and widely available marker of cardiometabolic risk. Given the increasing burden of cardiometabolic disorders among older adults, early identification of individuals at increased risk remains a major public health priority.

The TG/HDL‐c index also demonstrated a consistently high negative predictive value for prediabetes, diabetes, CVD, and cerebrovascular disease, suggesting that it may represent a useful screening tool to support cardiometabolic risk assessment in clinical practice. Our findings are consistent with previous studies evaluating TG‐based lipid indices. Baez‐Duarte et al. [20] reported excellent discriminatory performance of the TG/HDL‐c index for metabolic syndrome in a Mexican population, whereas Nosrati et al. [21] showed that the atherogenic index of plasma, calculated as the logarithm of the TG/HDL‐c ratio, was associated with poor glycemic control in patients with type 2 diabetes.

Compared with previous studies, higher optimal TG/HDL‐c cut‐off values were observed in our cohort of older adults. Similar age‐related differences have been reported in elderly populations [22]. These discrepancies likely reflect the unique metabolic profile associated with aging rather than differences in analytical performance alone. Aging is characterized by progressive insulin resistance, visceral adiposity, chronic low‐grade inflammation, and alterations in lipid metabolism that promote the accumulation of TG‐rich lipoproteins and reduce HDL functionality [23, 24].

In addition, differences in ethnicity, body fat distribution, lifestyle, socioeconomic conditions, and the prevalence of obesity, diabetes, and metabolic syndrome may further modify the relationship between the TG/HDL‐c index and cardiometabolic outcomes. Therefore, cut‐off values derived from younger or geographically distinct populations should not be directly extrapolated to older Colombian adults. These findings emphasize the importance of validating lipid‐based biomarkers across diverse populations and establishing population‐specific TG/HDL‐c thresholds to improve the identification of older adults at increased cardiometabolic risk.

Although LDL‐c remains the primary therapeutic target for cardiovascular prevention, substantial residual cardiometabolic risk persists despite optimal lipid‐lowering therapy. Consequently, increasing attention has been directed toward complementary biomarkers that better reflect insulin resistance, atherogenic dyslipidemia, and the complex metabolic alterations associated with aging [2, 11, 25]. Because the TG/HDL‐c index integrates two routinely available lipid parameters, it provides complementary information beyond conventional lipid measures and may complement existing cardiometabolic risk assessment and stratification in older adults.

The findings of this study have important implications for clinical practice, particularly in low‐ and middle‐income countries where access to advanced biomarkers may be limited. Because lipid profiles are routinely obtained during cardiovascular risk assessment, the TG/HDL‐c index can be calculated without additional laboratory testing or cost, making it an accessible and practical biomarker for routine care. The population‐specific cut‐off values identified in this study may facilitate early identification of older adults at increased risk of diabetes and metabolic syndrome, supporting timely lifestyle interventions, optimization of lipid and glycemic control, and referral for further evaluation when appropriate. Although prospective studies are needed to validate these cut‐off values and determine their prognostic performance, our findings support the potential incorporation of the TG/HDL‐c index as a complementary tool for cardiometabolic risk assessment, particularly in resource‐limited healthcare settings.

Importantly, cardiometabolic risk is influenced not only by biological factors but also by behavioral and social determinants of health. In a recent nationally representative study from our group [26], female sex, lack of health insurance, and physical inactivity were identified as important determinants of adverse lipid profiles in older Colombian adults. Together with the present findings, these results suggest that biological markers such as the TG/HDL‐c index should be interpreted within a broader framework that incorporates social and behavioral determinants. Such an integrated approach may facilitate earlier identification of high‐risk individuals and support more effective prevention strategies in aging populations.

This study has several limitations. First, its cross‐sectional design precludes establishing temporal or causal relationships between the TG/HDL‐c index and cardiometabolic outcomes. Although the TG/HDL‐c index was independently associated with the presence of diabetes and metabolic syndrome, the direction of these associations cannot be determined. Second, while the SABE survey provides a nationally representative sample of older Colombian adults, the findings may not be generalizable to populations with different demographic, ethnic, or epidemiological characteristics. Third, several variables, including lifestyle behaviors and medical history, were self‐reported and therefore may be subject to recall or misclassification bias. Furthermore, although the multivariable models adjusted for major confounders, residual confounding from unmeasured factors, such as medication use, detailed dietary patterns, or socioeconomic characteristics, cannot be excluded. Finally, the proposed cut‐off values were derived and evaluated within the same study population and require external validation in independent cohorts before routine clinical implementation. Despite these limitations, to our knowledge, this is the first nationally representative study to evaluate both the association and discriminatory performance of the TG/HDL‐c index for diabetes and metabolic syndrome in older Colombian adults. The large sample size and national representativeness strengthen the robustness and generalizability of our findings within Colombia and provide locally derived cut‐off values that may inform future research and clinical implementation. Future prospective studies should validate these cut‐off values in independent populations and evaluate the prognostic value of the TG/HDL‐c index for predicting long‐term cardiometabolic outcomes.

## Author Contributions

**Carolina Murgueitio Guzmán:** original idea, conceptualization, methods, data curation, formal analysis, writing – original draft, writing – review and editing. **Jesús Andrés Beltrán España:** conceptualization, methods, data curation, formal analysis, writing – review and editing. **Ángel Alberto García Peña:** original idea, conceptualization, methods, writing – review and editing, supervision, mentoring. **Edward Cáceres:** conceptualization, methods, data curation, formal analysis, writing – review and editing, supervision, mentoring. **Cándida Díaz‐Brochero:** conceptualization, data curation, writing (review and editing), supervision, mentoring. **Miguel Ángel Narváez Chaves:** methods, formal analysis, writing – original draft. **Luciano Villa Miranda:** methods, formal analysis, writing – original draft. All the other authors provided comments on the drafts of the manuscript and approved the final manuscript. The corresponding author attests that all listed authors meet authorship criteria and that no others meeting the criteria have been omitted. All authors approved the final manuscript and were responsible for deciding to submit it for publication.

## Funding

The authors have nothing to report.

## Conflicts of Interest

The authors declare no conflicts of interest.

## Transparency Statement

The lead/corresponding author (Carolina Murgueitio Guzmán) affirms that this manuscript is an honest, accurate, and transparent account of the study being reported; that no important aspects of the study have been omitted; and that any discrepancies from the study as planned (and, if relevant, registered) have been explained.

## Data Availability

The data that support the findings of this study are available in the supporting material of this article.

## References

[1] C. Qu , S. Liao , J. Zhang , et al., “Burden of Cardiovascular Disease Among Elderly: Based on the Global Burden of Disease Study 2019,” European Heart Journal – Quality of Care and Clinical Outcomes 10, no. 2 (March 2024): 143–153. 37296238 10.1093/ehjqcco/qcad033 PMC10904724

[2] C. E. Kosmas , S. Rodriguez Polanco , M. D. Bousvarou , et al., “The Triglyceride/High‐Density Lipoprotein Cholesterol (TG/HDL‐C) Ratio as a Risk Marker for Metabolic Syndrome and Cardiovascular Disease,” Diagnostics 13 (2023): 929. 36900073 10.3390/diagnostics13050929 PMC10001260

[3] E. Alegría , A. Cordero , M. Laclaustra , et al., “Prevalencia del síndrome metabólico en población laboral española: registro MESYAS,” Revista española de cardiología 58, no. 7 (2005): 797–806. 16022811

[4] F. Guerrero‐Romero , L. E. Simental‐Mendía , M. González‐Ortiz , et al., “The Product of Triglycerides and Glucose, a Simple Measure of Insulin Sensitivity. Comparison With the Euglycemic‐Hyperinsulinemic Clamp,” Journal of Clinical Endocrinology & Metabolism 95, no. 7 (2010): 3347–3351. 20484475 10.1210/jc.2010-0288

[5] L. Sánchez‐Íñigo , D. Navarro‐González , A. Fernández‐Montero , J. Pastrana‐Delgado , and J. A. Martínez , “The TyG Index May Predict the Development of Cardiovascular Events,” European Journal of Clinical Investigation 46, no. 2 (February 2016): 189–197. 26683265 10.1111/eci.12583

[6] R. Kones , “Molecular Sources of Residual Cardiovascular Risk, Clinical Signals, and Innovative Solutions: Relationship With Subclinical Disease, Undertreatment, and Poor Adherence: Implications of New Evidence Upon Optimizing Cardiovascular Patient Outcomes,” Vascular Health and Risk Management 9 (2013): 617–670. 24174878 10.2147/VHRM.S37119 PMC3808150

[7] A. Da Silva , A. P. S. Caldas , H. H. M. Hermsdorff , et al., “Triglyceride‐Glucose Index Is Associated With Symptomatic Coronary Artery Disease in Patients in Secondary Care,” Cardiovascular Diabetology 18, no. 1 (July 2019): 89. 31296225 10.1186/s12933-019-0893-2 PMC6625050

[8] I. Wakabayashi and T. Daimon , “Comparison of Discrimination for Cardio‐Metabolic Risk by Different Cut‐Off Values of the Ratio of Triglycerides to HDL Cholesterol,” Lipids in Health and Disease 18, no. 1 (July 2019): 156. 31351479 10.1186/s12944-019-1098-0 PMC6661090

[9] B. Park , D. H. Jung , H. S. Lee , and Y. J. Lee , “Triglyceride to HDL‐Cholesterol Ratio and the Incident Risk of Ischemic Heart Disease Among Koreans Without Diabetes: A Longitudinal Study Using National Health Insurance Data,” Frontiers in Cardiovascular Medicine 8 (2021): 716698. 34490378 10.3389/fcvm.2021.716698 PMC8418107

[10] A. Rozanski , J. A. Blumenthal , K. W. Davidson , P. G. Saab , and L. Kubzansky , “The Epidemiology, Pathophysiology, and Management of Psychosocial Risk Factors in Cardiac Practice,” Journal of the American College of Cardiology 45 (2005): 637–651. 15734605 10.1016/j.jacc.2004.12.005

[11] Y. Chen , Z. Chang , Y. Liu , et al., “Triglyceride to High‐Density Lipoprotein Cholesterol Ratio and Cardiovascular Events in the General Population: A Systematic Review and Meta‐Analysis of Cohort Studies,” Nutrition, Metabolism, and Cardiovascular Diseases 32, no. 2 (February 2022): 318–329. 10.1016/j.numecd.2021.11.005 34953633

[12] A. D. Mazza , “Insulin Resistance Syndrome and Glucose Dysregulation in the Elderly,” Clinics in Geriatric Medicine 24 (2008): 437–454. 18672181 10.1016/j.cger.2008.03.006

[13] F. Gomez , J. Corchuelo , C. L. Curcio , M. T. Calzada , and F. Mendez , “SABE Colombia: Survey on Health, Well‐Being, and Aging in Colombia – Study Design and Protocol,” Current Gerontology and Geriatrics Research 2016 (2016): 7910205. 27956896 10.1155/2016/7910205 PMC5124445

[14] D. Ortega‐Lenis and F. Mendez , “Survey on Health, Well‐Being and Aging. SABE Colombia 2015: Technical Report,” Colombia Medica 50, no. 2 (2019): 128–138. 31607769 10.25100/cm.v50i2.4557 PMC6774577

[15] International Diabetes Federation , Metabolic Syndrome (2006).

[16] M. Abbasian , M. Delvarianzadeh , H. Ebrahimi , and F. Khosravi , “Lipid Ratio as a Suitable Tool to Identify Individuals With MetS Risk: A Case‐ Control Study,” Diabetes & Metabolic Syndrome: Clinical Research & Reviews 11 (November 2017): S15–S19. 10.1016/j.dsx.2016.08.011 27575046

[17] R Core Team , R: A Language and Environment for Statistical Computing (R Foundation for Statistical Computing, 2022).

[18] G. Nie , S. Hou , M. Zhang , and W. Peng , “High TG/HDL Ratio Suggests a Higher Risk of Metabolic Syndrome Among an Elderly Chinese Population: A Cross‐Sectional Study,” BMJ Open 11, no. 3 (March 2021): e041519. 10.1136/bmjopen-2020-041519 PMC7986938 33753431

[19] A. Chauhan , A. Singhal , and P. Goyal , “TG/HDL Ratio: A Marker for Insulin Resistance and Atherosclerosis in Prediabetics or Not?,” Journal of Family Medicine and Primary Care 10, no. 10 (2021): 3700–3705. 10.4103/jfmpc.jfmpc_165_21 PMC8653431 34934668

[20] B. G. Baez‐Duarte , I. Zamora‐Ginez , S. O. Rodríguez‐Ramírez , K. Pesqueda‐Cendejas , and K. H. García‐Aragón , “TG/HDL Index to Identify Subjects With Metabolic Syndrome in the Mexican Population,” Gaceta Medica de Mexico 158, no. 5 (September 2022): 269–274. 10.24875/GMM.M22000693 36572023

[21] M. Nosrati , M. Safari , A. Alizadeh , M. Ahmadi , and A. Mahrooz , “The Atherogenic Index Log (Triglyceride/HDL‐Cholesterol) as a Biomarker to Identify Type 2 Diabetes Patients With Poor Glycemic Control,” International Journal of Preventive Medicine 12 (2021): 160. 35070193 10.4103/ijpvm.IJPVM_357_20 PMC8724629

[22] F. Saeedi , E. Baqeri , A. Bidokhti , M. Moodi , F. Sharifi , and S. M. Riahi , “Clinical Utility of Lipid Ratios as Potential Predictors of Metabolic Syndrome Among the Elderly Population: Birjand Longitudinal Aging Study (BLAS),” BMC Geriatrics 23, no. 1 (December 2023): 403. 37400781 10.1186/s12877-023-04040-8 PMC10316584

[23] T. Vilela de Sousa , A. M. R. Z. Cavalcante , N. X. Lima , et al., “Cardiovascular Risk Factors in the Elderly: A 10‐Year Follow‐Up Survival Analysis,” European Journal of Cardiovascular Nursing 22, no. 1 (January 2023): 43–52. 35574942 10.1093/eurjcn/zvac040

[24] A. K. Palmer and M. D. Jensen , “Metabolic Changes in Aging Humans: Current Evidence and Therapeutic Strategies,” Journal of Clinical Investigation. American Society for Clinical Investigation 132 (2022): e158451. 10.1172/JCI158451 PMC9374375 35968789

[25] C. Bonanad , A. Ayesta , S. García‐Blas , et al., “Factores de riesgo cardiovascular en el paciente mayor,” Revista Colombiana de Cardiologia 29, no. Suppl 3 (2022): S14–S19.

[26] P. L. Luz , D. Favarato , J. R. Faria‐Neto Junior , P. Lemos , and A. C. P. Chagas , “High Ratio of Triglycerides to HDL‐Cholesterol Predicts Extensive Coronary Disease,” Clinics 63, no. 4 (2008): 427–432. 18719750 10.1590/S1807-59322008000400003 PMC2664115

